# Supplementary figures and images for: Differences in the genome, methylome, and transcriptome do not differentiate isolates of Streptococcus equi subsp. equi from horses with acute clinical signs from isolates of inapparent carriers
Source: PLoS One. 2021 Jun 14;16(6):e0252804. doi: 10.1371/journal.pone.0252804 (PMC8202921; doi:10.1371/journal.pone.0252804)

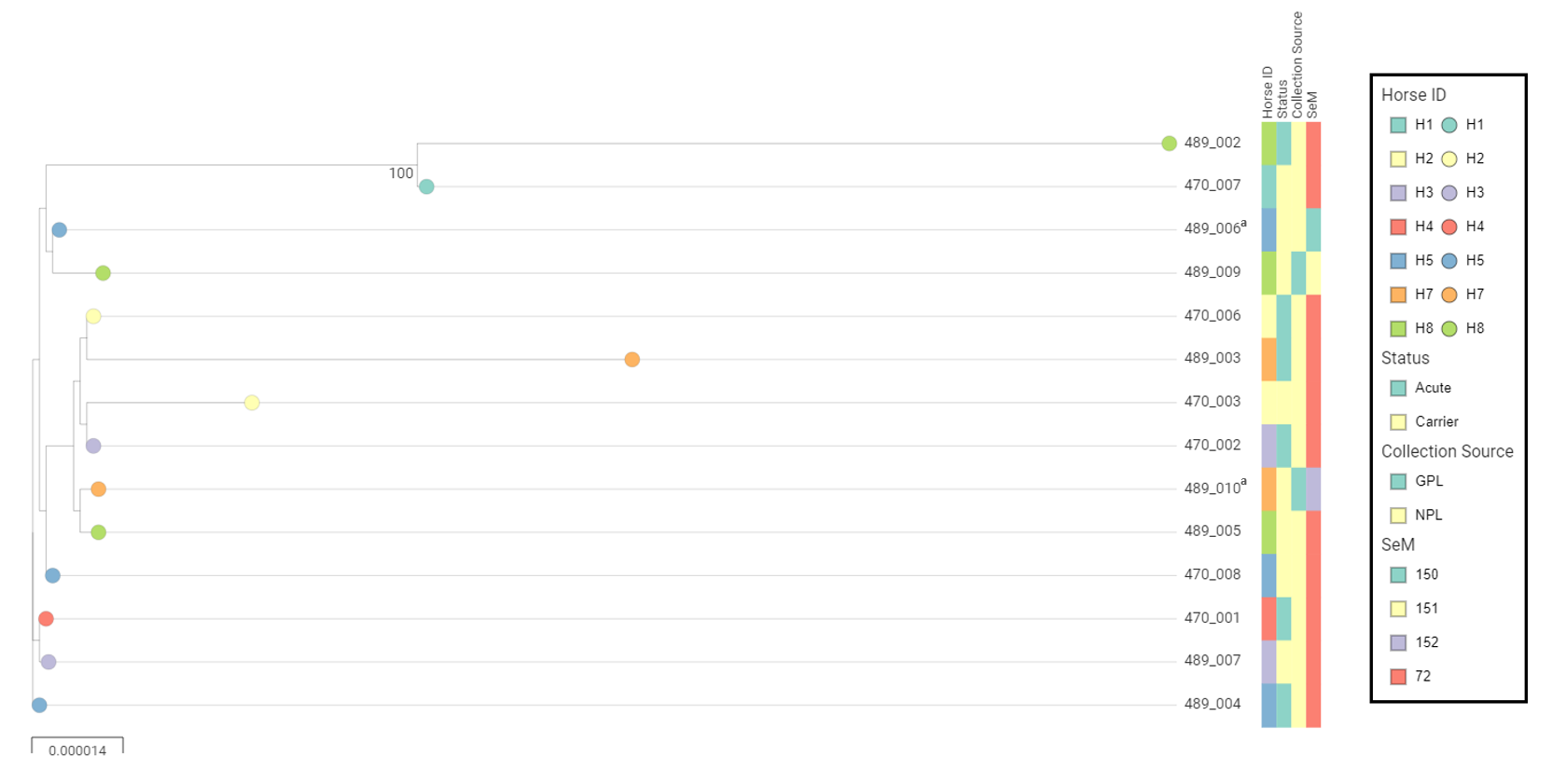

Supplement: S1 Fig — SEE isolates from the outbreak did not cluster by the individual horse from which the isolate was collected, but results demonstrate variation of isolates recovered from the same individual over time. aDenotes truncation in the SeM protein; GPL, Guttural pouch lavage; NL, Nasopharyngeal lavage; SeM, M-like protein. (TIF) [file pone.0252804.s001.tif]

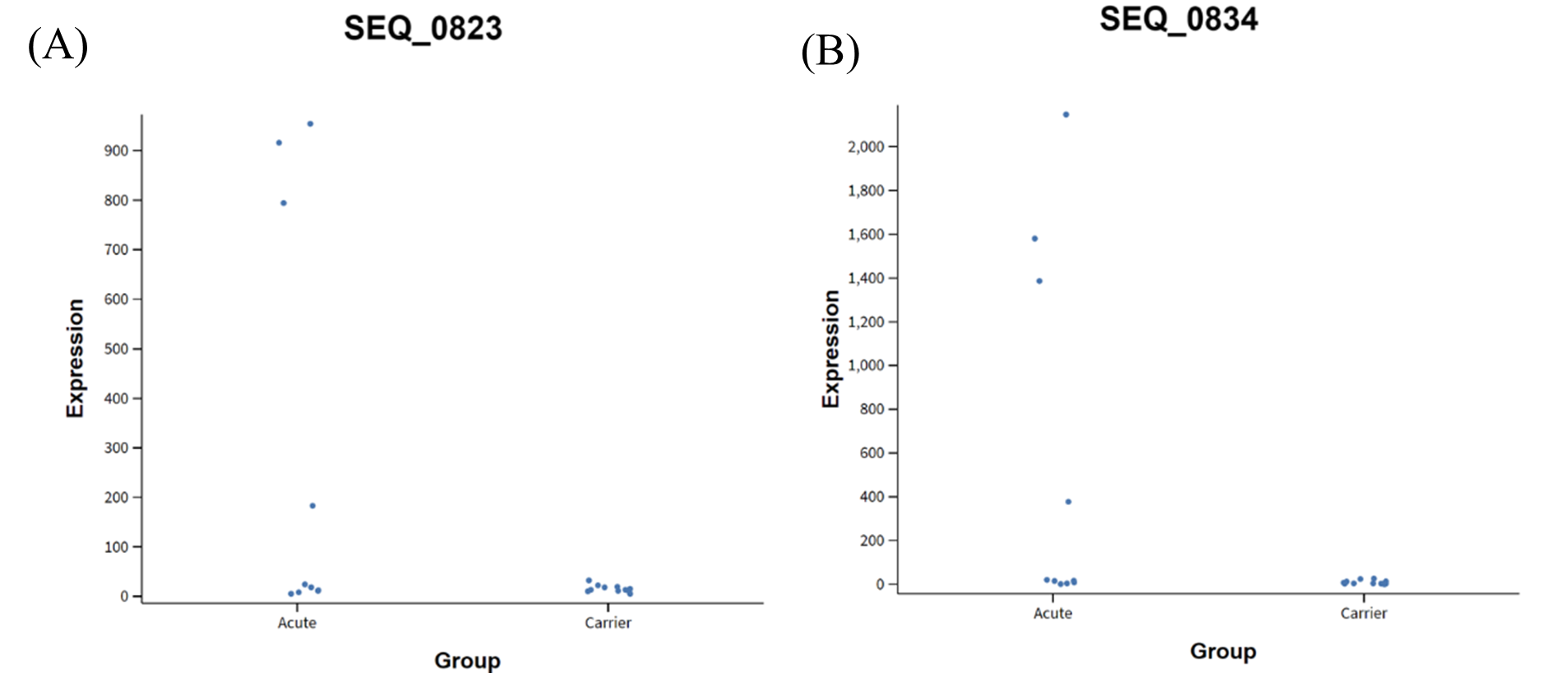

Supplement: S2 Fig — (A) Expression level (y-axis) of SEQ_0823 by disease presentation (x-axis). Only 3/10 of the acute SEE isolates had elevated expression levels. (B) Expression level (y-axis) of SEQ_0834 by disease presentation (x-axis). Only 3/10 of the acute SEE isolates had higher expression levels. (TIF) [file pone.0252804.s002.tif]
