# Supplementary material for: Differences in the genome, methylome, and transcriptome do not differentiate isolates of Streptococcus equi subsp. equi from horses with acute clinical signs from isolates of inapparent carriers
Source: PLoS One. 2021 Jun 14;16(6):e0252804. doi: 10.1371/journal.pone.0252804 (PMC8202921; doi:10.1371/journal.pone.0252804)
Supplement: Attachment — Submitted filename: Response Letter PLoS One May 19 2021.pdf [file pone.0252804.s012.pdf]

COLLEGE OF VETERINARY MEDICINE
& BIOMEDICAL SCIENCES

Department of Large Animal Clinical Sciences
4475 TAMU

TEXAS A&M UNIVERSITY

May 19, 2021

Prof. Yung-Fu Chang
Academic Editor
PLoS One

Subject: Revision of manuscript entitled, "**Differences in the Genome, Methylome, and Transcriptome Do Not Distinguish Isolates of *Streptococcus equi* subsp. *equi* from Horses with Acute Clinical Signs from Isolates of Inapparent Carriers**"

Dear Dr. Chang:

Thank you for your electronic message dated May 7, 2021 about the above-referenced manuscript. I also thank the reviewers who carefully considered our report. My coauthors and I have revised the report on the basis of the reviewers' comments. Below, we detail how each point raised by the reviewers was addressed in the revised report. The revised report with marked changes and an unmarked manuscript will be uploaded to the *PLoS One* website along with this response letter.

Comments from Reviewer 1:

Line 116: were any or all of the carriers examined by endoscopy to determine whether guttural pouch pathology was present? If so, it would be helpful to provide further information and to identify which carriers had endoscopically visible guttural pouch pathology. This point is picked up in the discussion (carrier isolates being obtained from animals with and without gross pouch abnormalities) and it would be useful to have more details provided.

AUTHORS' RESPONSE: We thank the reviewer for raising this important point. All of the carrier horses were examined endoscopically. Unfortunately, follow-up information with details was not determined for 6 of the 11 horses from Pennsylvania. We have revised the report to include a table summarizing these findings (new Supplemental Table 1), and also included some discussion of these findings.

Line 258: This is an interesting observation – could the authors speculate on possible reasons for this in the discussion? For example, whether this is potentially due to the Swedish isolates all being obtained from a single outbreak and over a shorter time frame?

AUTHORS' RESPONSE: We thank the reviewer for raising another great point. We think that the Swedish isolates had more similar (fewer) AGE because they were from a single outbreak. Interestingly, they were actually collected over a period of > 1 year, which demonstrates persistence of a single strain in horses without clinical signs for over 1 year (albeit with some variation including truncation of SeM). These isolates also had fewer contigs which might have contributed to more consistent results for isolates from the same outbreak. We have revised the

report to address these points.

Again, we appreciate your efforts and those of the reviewers. Please let us know if you have any questions or concerns regarding our revised report.

Sincerely,

Noah D. Cohen, VMD, MPH, PhD, Dipl. ACVIM-LA
Professor and Associate Department Head for Research and Graduate Studies
